# Measurement Properties of Canadian Agility and Movement Skill Assessment for Children Aged 9–12 Years Using Rasch Analysis

**DOI:** 10.3389/fpubh.2021.745449

**Published:** 2021-12-06

**Authors:** Jindong Chang, Liming Yong, Hai Yan, Jibing Wang, Naiqing Song

**Affiliations:** ^1^School of Physical Education, Southwest University, Chongqing, China; ^2^School of Mathematics and Statistics, Southwest University, Chongqing, China; ^3^Institute of Motor Quotient, Southwest University, Chongqing, China; ^4^Department of Kinesiology and Community Health, University of Illinois at Urbana-Champaign, Urbana, IL, United States; ^5^International College of Football, Tongji University, Shanghai, China; ^6^The Branch of the Collaborative Innovation Center of Assessment Toward Basic Education Quality, Southwest University, Chongqing, China

**Keywords:** agility, motor skills, assessment, Rasch analysis, CAMSA

## Abstract

The Canadian Agility and Movement Skill Assessment (CAMSA) was recently widely used to assess fundamental motor skills in children. Although the CAMSA is reported to be reliable and valid, its measurement properties are not clear. This study aimed to examine the measurement properties of the CAMSA in a sample of Chinese children using Rasch analysis. The study sample was from 1,094 children aged 9–12 years in Zunyi City, Guizhou Province. Descriptive data were analyzed using SPSS 24.0 software, and the dichotomous data were analyzed by Winsteps version 4.5.4 and Facets 3.67.1 software performing Rasch analysis. The present study investigated CAMSA measurement characteristics by Rasch analysis, including the reliability of the rating instrument, unidimensionality, item-fit statistics, and differential item functioning (DIF). Inter-rater reliability and retest reliability showed that the CAMSA had a good internal consistency. Rasch analysis indicated that the CAMSA was unidimensional, locally independent, and had a good item-fit-statistic. Additionally, the CAMSA displayed a good fit for the item separation index (12.50 > 2.0), as well as for item reliability (0.99 > 0.90). However, the item difficulty of the CAMSA did not fit well with personal ability, and a significant DIF was found across genders. In the Chinese children sample test, the CAMSA demonstrated appropriate goodness-of-fit validity and rater reliability. Thus, future research will explore item difficulty and person ability fit, as well as DIF across genders.

## Introduction

Children's fundamental motor skills (FMS) have long been described as a cornerstone of their physical activity, and they are typically classified into movement skills (e.g., running, sliding), object control skills (e.g., catching, kicking), and stability skills (e.g., balance) (1, 2). Proficiency in these skills has an important implication for children's healthy development (3, 4), yet numerous studies have indicated that the global rate of children's mastery is low (5). The development of FMS in childhood is crucial for the development of individuals as they develop through life. Therefore, the ideal FMS assessment tool should be provided during childhood, not only for diagnosing levels of motor skill development but also for targeting children's development in relation to motor skills.

Currently, numerous assessment tools are available internationally to assess children's FMS, such as TGMD (6, 7), MABC (8, 9), KTK (10, 11), and BOT (12, 13). A common feature of these assessment tools is that each movement is measured independently, and actions are not connected. These motor skill assessments accurately measure the completion of movements; however, the “real sport situation” is ignored, and the measurement results may deviate from an actual context (14). Contrary to these traditionally popular assessment tools, the Canadian Agility and Movement Skill Assessment (CAMSA) is the first international closed-loop motor skills assessment tool based on a series of combined movements (15). This assessment model is more closely matched to “real sport situations.” The term “real sport situation” refers to the fact that the movements in the assessment are coherent, continuous, and highly similar to the practical movement situation (16, 17). Additionally, the test results of this real sports can better reflect what is happening during children's movements. Initially, the CAMSA was developed to measure children's fundamental, complex, and integrated motor skills, with the primary purpose of diagnosing the level of motor development and identifying the risk of motor disorders in children (15). Since then, the CAMSA has been widely and concurrently used as a critical component of the Canadian Assessment of Physical Literacy (CAPL) (18). The Children's Physical Literacy Assessment application showed that the CAMSA assessment results were similar to real sport situations (19).

Numerous studies from Canada (20), Australia (2), the Netherlands (21), the United Kingdom (22), South Africa (23), and China (19) have explored the measurement properties of the CAMSA instrument. However, validity of the CAMSA test has only been reported in three studies to date (except the CAMSA development team) (2, 19, 20). Of these, Lander et al. showed that CAMSA skill scores had good concurrent validity (*r*_s_ = 0.68) and inter-rater retest reliability (ICC = 0.85) in an Australian study of early adolescent girls (2). Another study from Canada validated the reliability and validity of the PLAY*fun* using the CAMSA as a validity criterion (20). The PLAY*fun* is an instrument with similar functions to the CAMSA, and it is also used to measure children's motor proficiency. A study by Stearns et al. revealed moderate to large correlations between PLAYfun and CAMSA (*r* = 0.47–0.60) (20). Furthermore, another study from a sample of Chinese male children explored the validity of the CAMSA timing test in comparison to three commonly used agility tests (19).

In the original study, the validity of the CAMSA was determined using the expert Delphi method. ANOVA was used to examine age and gender differences, and paired *t*-tests were conducted to examine differences between the effects of footwear vs. no footwear, as well as indoors vs. outdoors (15). Despite some studies further confirming the validity of the CAMSA skills assessment instrument (2, 20), processing data from the assessment results are inadequate. In particular, as a dichotomous data variable for the results of each CAMSA item, Rasch analysis is an effective method for processing this category of data (14, 24, 25). However, there are currently no studies that have applied Rasch analysis to establish the validity of the CAMSA. Additionally, although the validity of the CAMSA has been demonstrated in both Canadian and Australian children, there is no reported evidence of its validity among Chinese children (14). Therefore, this present study aimed to validate the measurement properties of the CAMSA skills test instrument for Chinese children using Rasch analysis.

## Methods

### Participants

Clauser and Mazor stated that the sample size should not be too small if DIF analysis were to be conducted, with more than 500 participants at least (26). However, Mara and Angoff argued that, when the sample size was too large, the slightest difference in the test would be highly significant, increasing the probability of type I errors (27, 28). Therefore, the proposed collection of a sample of approximately 1,000 children aged 9–12 years is appropriate for this study. In addition, considering the gender balance of the participants, it would be sufficient for the gender ratio of children in each age group to be approximately equal. Therefore, at least 250 children should be included in each age group. Finally, a total of 1,094 children were recruited from six elementary schools in Zunyi, Guizhou Province, between October 8, 2019, and November 30, 2019. All children included should be physically non-disabled (no physical disabilities) and devoid of congenital disorders (e.g., heart disease).

### Instruments

**Figure 1:**
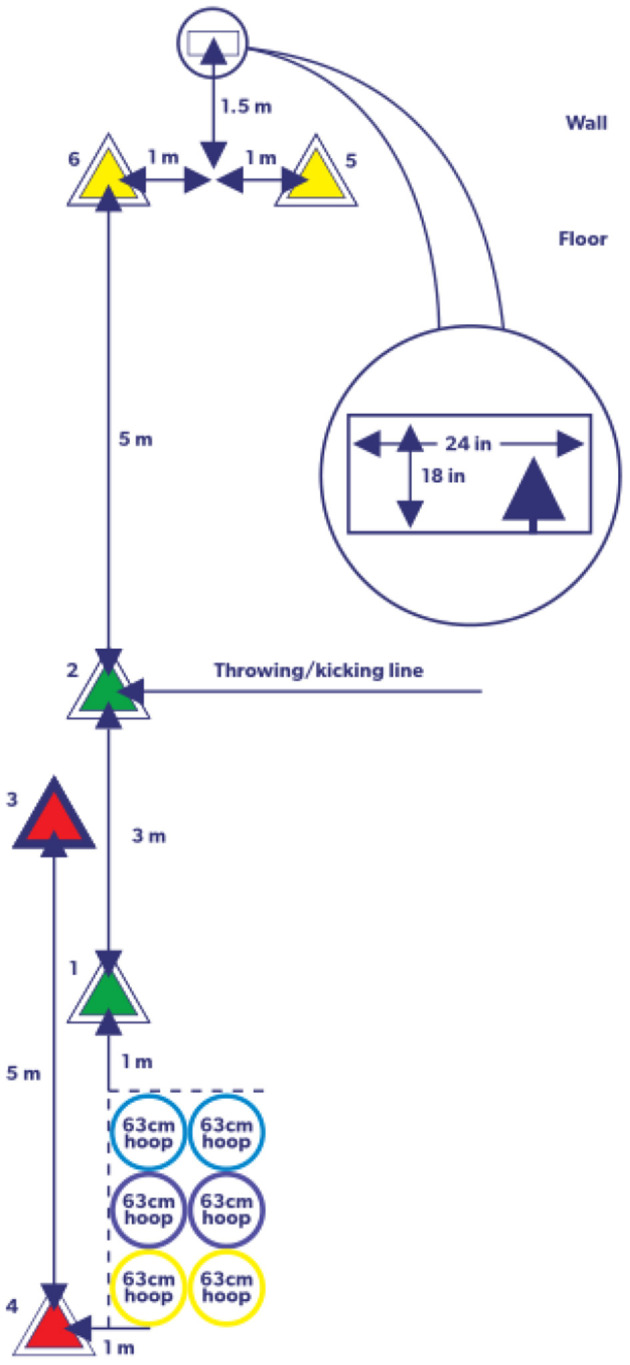
CAMSA layout (CAPL-2 edition).

The CAMSA is a movement capability assessment tool developed by Longmuir et al. for children aged 8–12 years (15). The CAMSA is used to assess children's fundamental movement skills and assess their capability to combine simple and complex movements. The CAMSA consists of seven movement items: two-foot jumping (2 points), sliding (3 points), catching (1 point), throwing (2 points), skipping (2 points), one-foot hop (2 points), and kicking (2 points) (Figure 1). The scoring points for each item are scored on a one-point scale (0–1). The skill score of the CAMSA is the total number of correctly completed skill movements and the total score ranges from 0 to 14 (15, 18). More details on CAMSA movement scoring can be found in the Canadian Assessment of Physical Literacy, Second Edition (CAPL-2, https://www.capl-ecsfp.ca) (29).

Anthropometric measurements were performed using the standard protocol of the “National Student Physical Fitness Standards” (NSPFS, 2014 revised version) (Ministry of Education of the People's Republic of China, 2014). The GJH1211 electronic tester was used to measure the height and weight of the participants, and the children were required to be barefoot and wear light clothing for the measurements. The test scale values were 0.1 cm for height and 0.1 kg for weight. The participants' body mass index (BMI) was calculated using BMI = height/weight^2^ (m/kg^2^). The BMI scoring criteria developed by the NSPFS were as follows. For boys: 9 years old (overweight 19.5–22.1, obesity ≥22.2, low weight ≤13.8); 10 years old (overweight 20.2–22.6, obesity ≥22.7, low weight ≤14.1); 11 years old (overweight 21.5–24.1, obesity ≥24.2, low weight ≤14.3); 12 years old (overweight 21.9–24.5, obesity ≥24.6, low weight ≤14.6). For girls: 9 years old (overweight 18.7–21.1, obesity ≥21.2, low weight ≤13.5); 10 years old (overweight 19.5–22.0, obesity ≥22.1, low weight ≤13.6); 11 years old (overweight 20.6–22.9, obesity ≥23.0, low weight ≤13.7); 12 years old (overweight 20.9–23.6, obesity ≥ 23.7, low body weight ≤14.1) (30).

### Procedures

This study was approved by the Ethics Committee of the Institute of Motor Quotient, Southwest University (IRB No: SWUIMQ20190516). The study protocol was guided by the guidelines of the International Declaration of Helsinki. All participants had obtained written permission from their parents/guardians in the study.

A total of 1,927 children from six schools went home with a CAMSA test presentation, a parent/guardian consent form, and a student demographic questionnaire. One week later, 1,366 signed parental consent forms and demographic questionnaires were received. Investigators screened the demographic information forms, and 61 children were excluded (21 children had physical defects and 40 children were assessed for rater consistency). Finally, the number of participants that actually completed the entire test and were included in this study came to 1,094.

The six raters underwent rigorous training in CAMSA testing and proficiently mastered the administration process and movement commands of the CAMSA assessment (15). Before formal testing, 40 students (five per age group, male and female) were randomly selected from one school and scored by six raters to ensure consistency in the assessment scale. Two raters were allocated to a team, with one team as primary and the other two as secondary in the scoring test. The three scoring teams took turns to be primary and secondary. The entire test was videotaped. After 1 week of the testing interval, six raters were asked to rate the video again. The data from the on-site test were used for inter-rater reliability testing, and the data from the two tests were examined for retest reliability. The formal test was only administered when the scores of the six raters met the consistency test criteria.

The children watched two demonstrations of the test movements before testing, in accordance with the CAMSA manual. The raters explained the movements and guidance words to the children during the video demonstrations. The children were given two practice trials and two scored trials. Scores on the best of the two tests were used as final scores. Testing was conducted outside and was suspended in the event of rain. Generally, the tests were performed in physical education classes or during extracurricular activities. One physical education teacher was scheduled to assist in the administration of the children, and the assessors were only responsible for the scoring and timing. During the assessment process, two raters alternated in rating and timing in accordance with the subgroups.

### Statistical Analysis

Descriptive statistical analyses were performed using SPSS 24.0 software to capture the demographic characteristics of the participants, including gender, age, and BMI. Rasch analysis was conducted to verify the construct validity of the CAMSA using WINSTEPS Version 4.5.4 software (31). Multi-faceted Rasch analysis was performed by using Facets Version 3.67.1 software (32, 33). To examine the measurement characteristics of CAMSA, a three-step procedure was performed following the CAMSA development guidelines.

First, inter-rater reliability and retest reliability were examined. Inter-rater reliability was evaluated using the multi-faceted Rasch model (MFRM). The MFRM is widely used to examine consistency among multiple raters. Zhu and Cole recommend a fit index infit and outfit (MnSq) criterion between 0.7 and 1.3 for motor skill assessment (34). We referred to Facets guidelines and adopted MnSq values between 0.7 and 1.3 for acceptable criteria in this study (35, 36). The intraclass correlation coefficient (ICC) was used to examine the rater's retest reliability (37). Wikstrom recommends the following criteria for ICC: scores of 0.9–1.0 for excellent; 0.80–0.89 for good; 0.7–0.79 for fair; and below 0.69 for poor (38).

Second, the Rasch residuals were tested for unidimensionality using principal component analysis (PCA). The PCA of the residuals was acceptable when the eigenvalue of the first factor extracted from the residuals was <2.0. Before PCA, the Rasch measurement dimension analysis involved examining point-measurement (PTMEA) biserial correlations and fit statistics (39). The measurement dimensions were confirmed to be devoid of negative PTMEA biserial correlations, and the fit statistics were stable, with no sudden high or low fits.

The difficulty independence assumption was tested using latent parallel analysis (LPA). Once the CAMSA showed a single dominant measurement structure, we performed item-level analyses using the Rasch model. Rasch analysis mainly includes item-fit statistics and differential item functioning (DIF) (37). The two goodness-of-fit statistics were used to examine the fit of the item model, namely infit and outfit (MnSq) (40). In the Rasch model, items are weighted, along with a linear logistic function, in accordance with their level of difficulty. The ratio of observed variance to expected variance will be 1.0 if an item fits this linear function exactly. Mean square values significantly more than 1.0 indicate model underfit, in contrast to values <1.0, which indicate model overfit. Wright and Linuck showed that the acceptable range of MnSq values is between 0.5 and 1.5 (41). Subsequently, some scholars recommend that a standard of MnSq values between 0.6 and 1.4 is better (42–44). Additionally, Bond and Fox argue that MnSq values between 0.7 and 1.3 are more accurate (45). Therefore, MnSq values between 0.7 and 1.3 were used in this study.

In addition, item reliability was assessed in terms of “separation” (G), which was considered to be the ratio of the true distribution of measurements to their measurement error (46). Item separation indices more significant than 2.0 are considered to be good. A related indicator is the reliability of these separation indices, with coefficients ranging from 0 to 1; a coefficient of 0.80 is considered good and 0.90 is considered excellent (46).

## Results

### Demographic Characteristics

Table 1 Presents the demographic characteristics of the participants. A total of 49.8% of male and 50.2% of female subjects completed all tests. The BMIs of all subjects showed that 81.6% were normal, 17.1% were overweight or obese, and 1.3% were underweight. The family status of the subjects showed that 16.8% of the children had lived without their parents for a long time, while only one parent accompanied 23.8% of the participants and both parents accompanied 59.4%. A total of 60.3% of the subjects were from urban areas and 39.7% were from rural areas.

**Table 1:** Demographic characteristics of the participants.

**Demographic variables, *n* (%)**	**Overall**	**Boys**	**Girls**
Gender	1,094 (100)	545 (49.8)	549 (50.2)
**Age, years**			
9	271 (24.8)	125 (22.9)	146 (26.6)
10	265 (24.2)	141 (25.9)	124 (22.6)
11	269 (24.6)	137 (25.1)	132 (24.0)
12	289 (26.4)	142 (26.1)	147 (26.8)
**BMI**			
Normal	893 (81.6)	437 (80.2)	456 (83.1)
Overweight	115 (10.5)	57 (10.5)	58 (10.6)
Obesity	72 (6.6)	41 (7.5)	31 (5.6)
Underweight	14 (1.3)	10 (1.8)	4 (0.7)
**Race**			
Han Nationality	1,033 (94.4)	505 (92.7)	528 (96.2)
Minorities	61 (5.6)	40 (7.3)	21 (3.8)
**Family**			
Lived without parents	184 (16.8)	91 (16.7)	92 (16.8)
Lived with one parent	260 (23.8)	134 (24.6)	126 (23.0)
Lived with parents	650 (59.4)	319 (58.5)	331 (60.3)
**Urban/Rural**			
Urban	660 (60.3)	327 (60.0)	333 (60.7)
Rural	434 (39.7)	218 (40.0)	216 (39.3)

### Inter-rater and Retest Reliability

Table 2 presents the results of the inter-rater reliability and retest reliability. The inter-rater reliability results showed that R4 is the strictest and R1 is the loosest. The infit MnSq values of the raters ranged between 0.99 and 1.05, which fit well with the acceptable standard interval of 0.7–1.3. Item separation coefficients of zero and far less than two indicated that inter-rater differences could not be effectively distinguished. In other words, there was no significant variability among raters. The ICC results showed that the retest reliability of the six raters ranged from 0.979 to 0.987, indicating that raters were skilled in the scoring rules.

**Table 2:** The results of the inter-rater reliability and retest reliability.

**Rater**	**Model**		**Infit**		**Outfit**		**ICC**
	**Measure**	**S.E**.	**MnSq**	**ZStd**	**MnSq**	**ZStd**	
R4	0.08	0.13	1.02	0.4	0.92	−0.3	0.987
R2	0.04	0.13	0.99	−0.2	0.88	−0.6	0.984
R5	0.01	0.13	1.05	0.9	1.29	1.5	0.984
R3	−0.01	0.13	1.00	0	0.89	−0.5	0.979
R6	−0.04	0.13	1.00	0	0.90	−0.5	0.982
R1	−0.08	0.13	0.99	−0.2	0.88	−0.6	0.971

*RMSE (model).13, Adj (True) S.D.00, Separation.00, Strata.33, Reliability.00*.

### Rasch Analysis

The results for the measurement dimensions were confirmed to be without negative correlation of PTMEA (*r* = 0.14–0.46), and the fit statistics were stable (infit MnSq = 0.90–1.12), with no abrupt high or low fit (Table 3). The PCA of the residuals indicated that the eigenvalue of the first factor extracted from the residuals was 1.7 <2, indicating that the model was consistent with unidimensionality. The correlation between the residuals of the items was not significant (*r* = 0.01–0.21). Therefore, the local independence assumption was not violated for any item.

**Table 3:** Item measure and fit statistics.

**Item**	**Model**		**Infit**		**Outfit**		**PTMEASUR-AL**	
	**Measure**	**S.E**.	**MnSq**	**ZStd**	**MnSq**	**ZStd**	**CORR**.	**EXP**.
CS07	2.73	0.08	1.09	2.16	1.21	3.13	0.27	0.37
CS12	1.14	0.07	1.00	−0.04	1.00	−0.03	0.39	0.38
CS08	1.10	0.07	1.02	1.07	1.01	0.35	0.36	0.38
CS10	0.53	0.07	0.90	−3.64	0.85	−3.72	0.46	0.36
CS09	0.11	0.07	0.92	−2.42	0.95	−0.97	0.41	0.34
CS13	0.06	0.07	1.01	0.26	1.01	0.23	0.32	0.33
CS14	−0.24	0.08	1.05	1.10	1.10	1.35	0.26	0.31
CS04	−0.27	0.08	0.95	−1.08	0.90	−1.46	0.36	0.31
CS11	−0.33	0.08	0.98	−0.48	0.92	−1.05	0.33	0.31
CS03	−0.41	0.08	0.94	−1.13	0.85	−2.03	0.37	0.30
CS05	−0.43	0.08	1.00	0.05	0.96	−0.42	0.30	0.30
CS06	−0.70	0.09	1.03	0.57	1.06	0.65	0.25	0.28
CS01	−0.72	0.09	1.12	1.97	1.34	3.24	0.14	0.28
CS02	−2.56	0.18	1.01	0.13	1.00	0.08	0.13	0.14
Mean	0.00	0.09	1.00	−0.10	1.01	0.00		
P.SD	1.15	0.03	0.06	1.50	0.13	1.80		

The infit and outfit MnSq values for all items were within the standard interval of 0.7–1.3, except for item CS01, which exhibited marginal misfit (outfit MnSq = 1.34). Item CS07 was the most difficult (2.73 logits), while item CS02 was the easiest (−2.56 logits). The results indicated that the item separation index was strong (G = 12.50), being well-above the criterion of 2.0. Item separation reliability was also excellent (*r* = 0.99).

**Figure 2:**
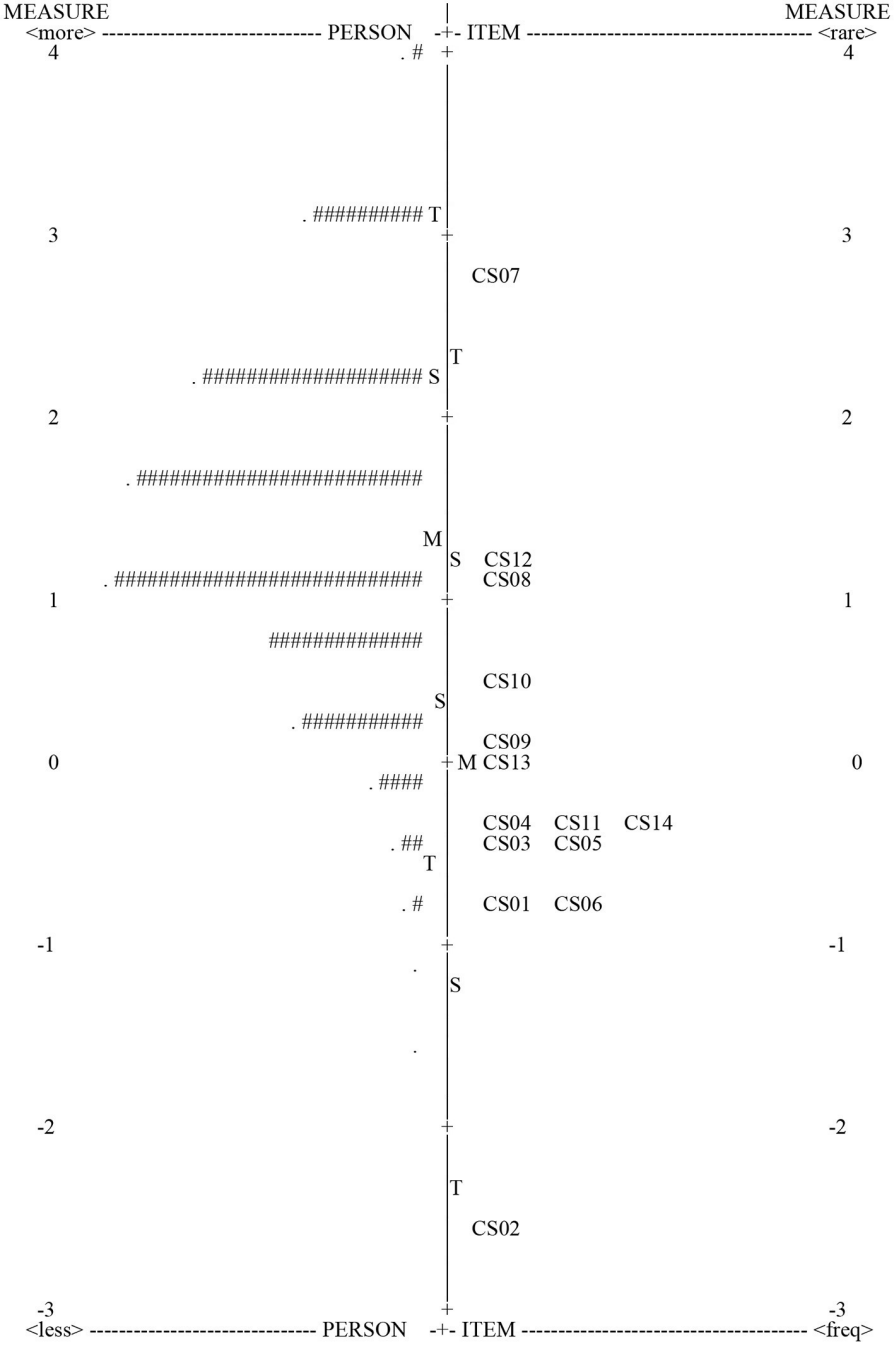
The map of person-item response difficulty locations. Each “#” in the PERSON column is 9 PERSON; each “.” is 1 to 8. +M, item mean; M, person mean; S, 1 SD from the mean; T, 2 SD from the mean.

Figure 2 located both items (difficulty levels) and persons (distribution of person ability) on the same continuum of fundamental movement skill. The figure indicated that the subjects' fundamental movement skill levels ranged from −1.58 to 4.51 logits, and the range of item response difficulty (−2.56 to 2.73 logits) was less than the subjects' fundamental movement skill levels; however, the range of item response difficulty covered 90.7% (*n* = 991) of the subjects. In addition, only 0.9% (*n* = 10) of the subjects scored in the highest scoring zone (raw score = 14), and 0.6% (*n* = 2) scored in the lowest zone (raw score = 3). Thus, there was no significant floor or ceiling effect. Also, Figure 2 showed that some test items have similar levels of difficulty (e.g., items CS03, CS11, and CS14), but most items target different levels of fundamental motor skill.

The DIF analysis revealed four items falling outside the criteria across the age dimension (Table 4). In particular, three items were found between the ages of 11 and 12 (CS06 DIF contrast = −0.62, *p* = 0.03; CS07 DIF contrast = −0.53, *p* = 0.01; CS11 DIF contrast = −0.55, *p* = 0.03) (14). One item was identified between the ages of 10 and 11 (CS01 DIF contrast = −0.59, *p* = 0.03). The DIF test demonstrated that the four items CS01, CS06, CS07, and CS11, were responded to differently by subjects of different ages (Table 4). For the CAMSA, this means that the measurement invariance of the items varies across age groups. In addition, the DIF analysis found that ten items did not fit the criteria across genders, indicating that there were measurable differences in the difficulty of the ten items between genders (Table 5).

**Table 4:** Differential item functioning (DIF = Age).

**PERSON**	**DIF**	**JOINT**	**Rasch-Welch**			**Mantel-Haenszel**		**ITEM**
**CLASS**	**CONTRAST**	**S.E**.	***t***	***df***	***Prob*.**	**Chi-squ**	***Prob*.**	
9 vs. 10	0.37	0.26	1.42	510	0.16	1.89	0.17	CS01
9 vs. 10	−0.40	0.52	−0.77	528	0.44	0.62	0.43	CS02
9 vs. 10	0.01	0.23	0.06	527	0.95	0.04	0.84	CS03
9 vs. 10	−0.04	0.22	−0.19	528	0.85	0.10	0.76	CS04
9 vs. 10	−0.02	0.22	−0.11	527	0.91	0.00	0.95	CS05
9 vs. 10	0.11	0.24	0.46	523	0.64	0.03	0.86	CS06
9 vs. 10	0.04	0.23	0.17	531	0.86	0.54	0.46	CS07
9 vs. 10	0.13	0.19	0.70	531	0.49	0.39	0.53	CS08
9 vs. 10	−0.13	0.20	−0.63	530	0.53	0.86	0.35	CS09
9 vs. 10	−0.02	0.19	−0.13	530	0.90	0.02	0.89	CS10
9 vs. 10	−0.24	0.22	−1.09	531	0.28	1.33	0.25	CS11
9 vs. 10	0.10	0.19	0.55	531	0.58	0.13	0.72	CS12
9 vs. 10	−0.07	0.20	−0.35	529	0.73	0.00	0.95	CS13
9 vs. 10	−0.09	0.22	−0.40	529	0.69	0.01	0.93	CS14
10 vs. 11	−0.59	0.27	−2.20	518	0.03^*^	2.58	0.11	CS01
10 vs. 11	−0.11	0.50	−0.22	525	0.83	0.00	1.00	CS02
10 vs. 11	−0.46	0.23	−1.98	523	0.05	4.29	0.04^*^	CS03
10 vs. 11	−0.32	0.23	−1.40	526	0.16	2.80	0.09	CS04
10 vs. 11	0.02	0.24	0.10	526	0.92	0.01	0.92	CS05
10 vs. 11	0.48	0.28	1.72	506	0.09	2.61	0.11	CS06
10 vs. 11	0.12	0.21	0.54	525	0.59	0.19	0.67	CS07
10 vs. 11	0.21	0.19	1.09	526	0.27	1.26	0.26	CS08
10 vs. 11	−0.05	0.21	−0.25	526	0.80	0.19	0.66	CS09
10 vs. 11	0.21	0.20	1.06	526	0.29	0.71	0.40	CS10
10 vs. 11	0.38	0.24	1.57	517	0.12	1.76	0.18	CS11
10 vs. 11	−0.13	0.19	−0.67	526	0.51	0.12	0.73	CS12
10 vs. 11	0.23	0.22	1.07	524	0.29	0.98	0.32	CS13
10 vs. 11	−0.20	0.22	−0.91	526	0.37	0.47	0.49	CS14
11 vs. 12	0.06	0.26	0.25	546	0.81	0.72	0.40	CS01
11 vs. 12	−0.11	0.53	−0.20	546	0.84	0.04	0.84	CS02
11 vs. 12	0.38	0.24	1.58	541	0.12	2.12	0.15	CS03
11 vs. 12	0.23	0.23	1.01	545	0.31	1.27	0.26	CS04
11 vs. 12	0.07	0.25	0.27	546	0.79	0.01	0.94	CS05
11 vs. 12	−0.62	0.29	−2.16	527	0.03^*^	2.78	0.10	CS06
11 vs. 12	−0.53	0.21	−2.53	546	0.01^*^	3.62	0.06	CS07
11 vs. 12	−0.26	0.19	−1.38	543	0.17	1.14	0.28	CS08
11 vs. 12	0.23	0.22	1.05	546	0.30	0.32	0.57	CS09
11 vs. 12	0.27	0.21	1.29	546	0.20	0.24	0.62	CS10
11 vs. 12	−0.55	0.24	−2.24	534	0.03^*^	5.47	0.02^*^	CS11
11 vs. 12	0.24	0.19	1.28	545	0.20	0.99	0.32	CS12
11 vs. 12	−0.03	0.23	−0.13	546	0.90	0.01	0.93	CS13
11 vs. 12	0.30	0.24	1.29	544	0.20	1.03	0.31	CS14

* *p < 0.05*.

**Table 5:** Differential item functioning (DIF = gender).

**PERSON**	**DIF**	**JOINT**	**Rasch-Welch**			**Mantel-Haenszel**		**Size**	**Item**
**CLASS**	**CONTRAST**	**S.E**.	***t***	***d.f*.**	***Prob*.**	**Chi-squ**	***Prob*.**	**CUMLOR**	
1 vs. 2	0.41	0.18	2.27	INF	0.02^*^	2.76	0.10	0.32	CS01
1 vs. 2	0.83	0.39	2.15	INF	0.03^*^	2.98	0.08	0.74	CS02
1 vs. 2	0.26	0.17	1.55	INF	0.12	3.01	0.08	0.32	CS03
1 vs. 2	0.16	0.16	0.99	INF	0.32	1.32	0.25	0.20	CS04
1 vs. 2	−0.15	0.17	−0.87	INF	0.38	0.71	0.40	−0.16	CS05
1 vs. 2	−0.43	0.18	−2.33	INF	0.02^*^	4.89	0.03^*^	−0.42	CS06
1 vs. 2	−0.51	0.16	−3.24	INF	0.00^*^	10.79	0.00^*^	−0.51	CS07
1 vs. 2	−1.03	0.14	−7.57	INF	0.00^*^	54.65	0.00^*^	−1.00	CS08
1 vs. 2	0.60	0.15	4.01	INF	0.00^*^	17.67	0.00^*^	0.68	CS09
1 vs. 2	0.52	0.14	3.77	INF	0.00^*^	18.10	0.00^*^	0.65	CS10
1 vs. 2	0.45	0.16	2.77	INF	0.01^*^	7.66	0.01^*^	0.47	CS11
1 vs. 2	0.67	0.13	5.08	INF	0.00^*^	24.27	0.00^*^	0.68	CS12
1 vs. 2	−0.29	0.15	−1.93	INF	0.05	3.23	0.07	−0.28	CS13
1 vs. 2	−0.90	0.17	−5.37	INF	0.00^*^	27.70	0.00^*^	−0.86	CS14

* *p < 0.05*.

## Discussion

The CAMSA is a fundamental movement skills assessment tool for children, but its measurement properties have yet to be thoroughly investigated. The purpose of this study was to investigate the measurement properties of the CAMSA using a Rasch model. This study was the first to use the Rasch model to investigate the CAMSA measurement properties, including item unidimensionality, local independence, item-fit, and differential item functioning (DIF) (47, 48). Overall, the CAMSA displayed adequate inter-rater reliability, retest reliability, internal consistency, and structural validity.

As expected, a multi-faceted Rasch model to test the consistency of the six raters showed good inter-rater reliability for CAMSA. The rater re-rated after a 1-week interval also demonstrated a positive rater retest reliability. Compared to the retest reliability of rater skills reported by CAMSA developers (ICC = 0.69) (15) and applications in Australia (ICC = 0.85) (2), the rater levels in this study (ICC = 0.979–0.987) were superior to the former. The rater reliability test demonstrated the necessity and validity of the raters' training and provided strong evidence that the CAMSA scoring rules are clear and valid. The establishment of inter-rater reliability and retest reliability assured the quality of CAMSA data collection.

Rasch analysis demonstrated that the CAMSA test is unidimensional, with no items violating the local independence assumption, and it is generally a hierarchical and well-developed assessment instrument (46). Analysis of the fit statistics indicated that the items were well-fitted, except for one item (CS01) outside the fitting border in the outfit MnSq. However, some studies suggested that infit values were more stable than outfit values in the item-fit statistics (46). The item CS01 infit MnSq of 1.12 is a trustworthy fit value. Therefore, the overall fit statistics of the CAMSA are regarded as good.

The person-item Wright map (Figure 2) shows that item difficulty did not match well with person-ability. With 38.4% of persons between 1 and 2.5 logits, no matching assessment items were found in this interval. In contrast, in the interval of −1 and 0 logits, eight items (57.1%) distinguished only 8.3% of the person-ability. Overall, the test results indicated that the item difficulty of the CAMSA test was low and the person ability was high (49). Therefore, the item difficulty of the CAMSA test struggles to differentiate individual abilities precisely (50). In general, for test results with low item difficulty and high personnel competency, adjusting item difficulty or test sequence is used to calibrate the assessment tool. Since the CAMSA is a closed-ended test instrument, adjusting the action sequence may change the properties of the original test structure. As such, a more appropriate method may be to increase the difficulty of the test items. In particular, in item-intensive locations (−1 to 0 logits), adjusting item difficulty may be a better strategy.

The differential item functioning analysis showed that four items (9.5%) existed DIF by the age factor. However, ten items (71.4%) were significantly DIF by the gender factor. The DIF items that existed by the age factor were mainly between the ages of 11 and 12, while no items between the ages of 9 and 10 years existed significantly in DIF. In terms of the age factor, the number of DIF items was greater in the older group than in the younger group, indicating that the low difficulty of the items may have led to a decrease in the differentiation of the older group, and thus to the DIF. Furthermore, the DIF was present in 71.4% of the items in the gender factor. The difference in movement test results across genders is inevitable in terms of physical and physiological interpretation. However, this also suggests that we should be cautious in interpreting differences between genders when using CAMSA test results.

First, although the validity of the CAMSA skills test fitting was good, poor matching of personal ability to item difficulty may result in differences in CAMSA discrimination. Second, the differential item functioning across genders prompted us to be cautious about comparing the results between men and women. Third, although the CAMSA is a well-tested assessment of “real sport” motor skills, there are still many agility motor skills (e.g., bilateral coordination, dynamic balance) that may not be assessed. Therefore, we need to improve the validity of the CAMSA measurements by increasing movement difficulty, adjusting movement scoring criteria, and enhancing the match between the difficulty of the instrument's movements and personal ability. This is a task for future studies. Furthermore, we recommend and encourage researchers and elementary physical education teachers to use the CAMSA to replace the traditional assessment tool of fundamental movement skills.

## Conclusions

The present study is the first to analyze the measurement properties of the CAMSA using the Rasch model. The CAMSA, as a closed-loop measure of fundamental movement skills in children, demonstrated good unidimensionality, local assumption independence, and item-fit statistics. The inter-rater reliability and retest reliability revealed that the CAMSA was internally consistent. However, there were significant differences in its person–item fit matching and across genders in relation to differential item functioning.

## Data Availability Statement

The raw data supporting the conclusions of this article will be made available by the authors, without undue reservation.

## Ethics Statement

The studies involving human participants were reviewed and approved by the Scientific and Ethics Committee of Institute of Motor Quotient, Southwest University (IRB NO. SWUIMQ20190605). Written informed consent to participate in this study was provided by the participants' legal guardian/next of kin.

## Author Contributions

JC and LY: data collection. JC and HY: data analysis. JC, JW, and NS: conception and design. JC, LY, HY, and JW: writing the manuscript and revision. All authors contributed to the article and approved the submitted version.

## Funding

This study was funded by Humanities and Social Sciences Project of Ministry of Education (17YJC890020), National Social Science Fund (15CTY011), Postgraduate Education Research Project of Tongji University (2021GL15), the Fundamental Research Funds for the Central Universities of Southwest University (SWU1709240), the Education and Teaching Reform Research Project of Southwest University (2021JY071), and Chongqing Educational Science Planning Project (2020-GX-257).

## Conflict of Interest

The authors declare that the research was conducted in the absence of any commercial or financial relationships that could be construed as a potential conflict of interest.

## Publisher's Note

All claims expressed in this article are solely those of the authors and do not necessarily represent those of their affiliated organizations, or those of the publisher, the editors and the reviewers. Any product that may be evaluated in this article, or claim that may be made by its manufacturer, is not guaranteed or endorsed by the publisher.
